# Supplementary material for: Effects of transient, persistent, and resurgent sodium currents on excitability and spike regularity in vestibular ganglion neurons
Source: Front Neurol. 2024 Nov 18;15:1471118. doi: 10.3389/fneur.2024.1471118 (PMC11608953; doi:10.3389/fneur.2024.1471118)

Supplementary Figure

Figure S1 *Comparison of VGN responses to model VGN simulations.*

(A – C) (reproduced from Fig. 5). Averaged recorded APs (A), corresponding mean phase plane plots (B), and exemplar step-evoked firing patterns (C). **(D)** Model-generated APs based on APs from (A). **(E)** Phase-plane plots for simulated APs from (D). Simulated APs have smaller peak rates of depolarization. **(F)** Step-evoked firing patterns of model VGNs capture key features of exemplar data in (C).

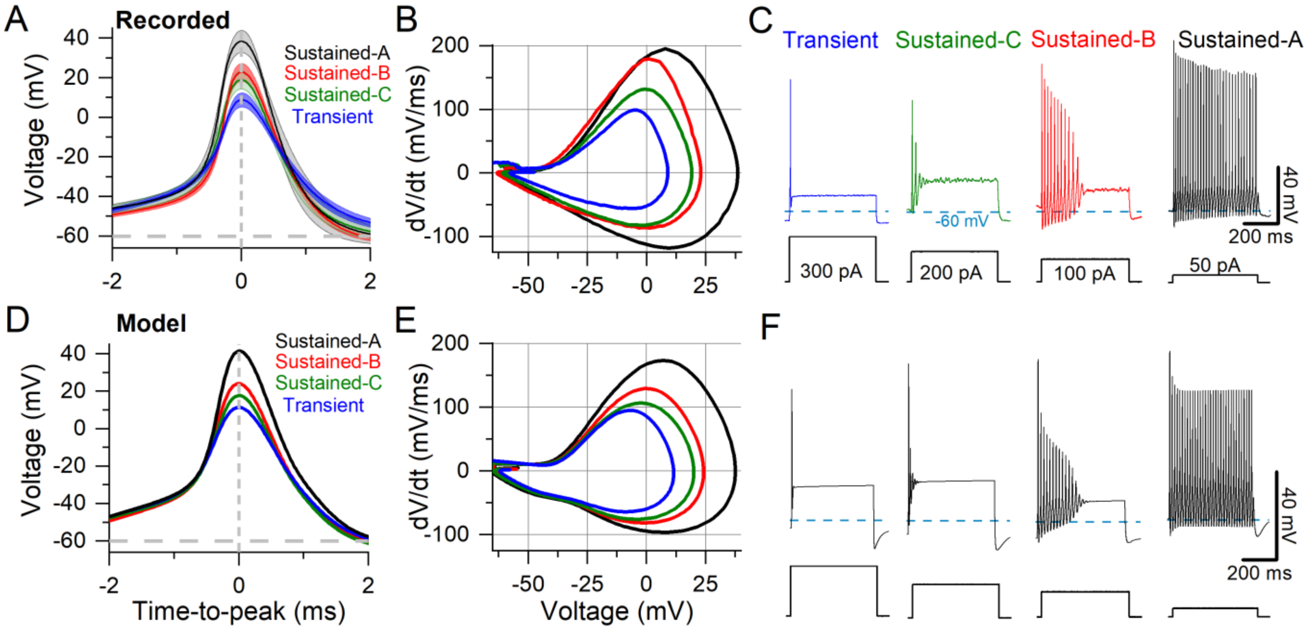

Supplement: Supplementary file 1 [file Image_1.pdf]
