# Supplementary material for: Effects of transient, persistent, and resurgent sodium currents on excitability and spike regularity in vestibular ganglion neurons
Source: Front Neurol. 2024 Nov 18;15:1471118. doi: 10.3389/fneur.2024.1471118 (PMC11608953; doi:10.3389/fneur.2024.1471118)

Supplementary Figure

Figure S2 ***Shape of simulated EPSCs.***

Simulated excitatory postsynaptic currents (EPSCs) used to evoke spiking in our isolated-soma models were based on spontaneous vestibular synaptic potentials (s_V) recorded at room temperature in mouse calyces (López-Ramirez, González-Garrido and Eatock, unpublished data). EPSC shapes previously tested in Hight and Kalluri (2016) (s1, s2, and s3) are shown for comparison.

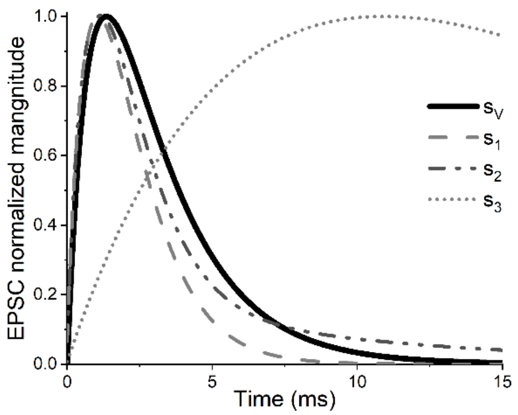

Supplement: Supplementary file 2 [file Image_2.pdf]
