# Supplementary figures and images for: Effects of transient, persistent, and resurgent sodium currents on excitability and spike regularity in vestibular ganglion neurons
Source: Front Neurol. 2024 Nov 18;15:1471118. doi: 10.3389/fneur.2024.1471118 (PMC11608953; doi:10.3389/fneur.2024.1471118)

## Supplementary Figure

**Figure S3** *Changes in firing patterns and with age.*

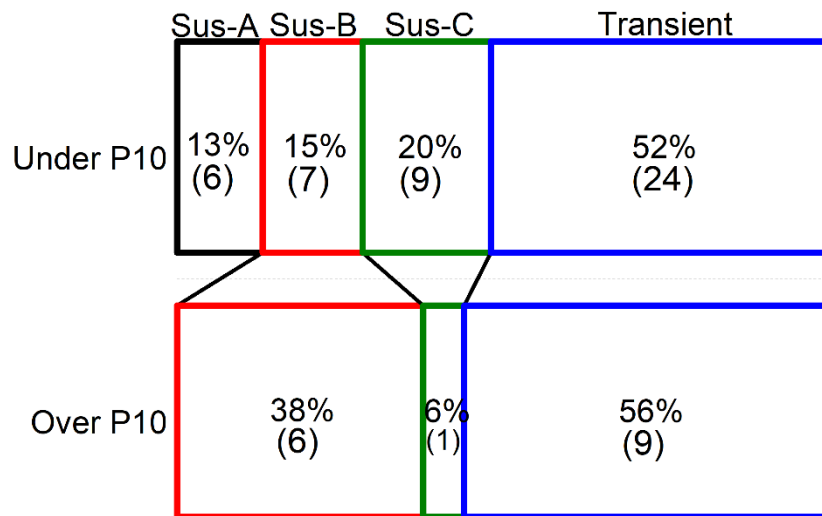

Supplement: Supplementary file 3 [file Image_3.pdf]
