# Supplementary material for: Effects of transient, persistent, and resurgent sodium currents on excitability and spike regularity in vestibular ganglion neurons
Source: Front Neurol. 2024 Nov 18;15:1471118. doi: 10.3389/fneur.2024.1471118 (PMC11608953; doi:10.3389/fneur.2024.1471118)

# Supplementary Figure

**Figure S4** ***Reducing $Na_V$ current modes replicates 4,9-ah-TTX block in model sustained-A and transient VGNs.***

**(A & B)** Firing patterns for a sustained-A (A) and transient (B) mVGNs. Reducing (red) I-$Na_V$T by 70% and I-$Na_V$R and I-$Na_V$P by 90% replicates results obtained with application of 4,9-ah-TTX in sustained-A mVGNs (see Fig. 6): increase in current threshold, reduction in spike height and spike rate, and slight hyperpolarization in $V_{rest}$.

**(B)** Transient mVGNs had reduced spike height and (not shown) greater current threshold.

**(C)** $Na_V$P and $Na_V$T drive hyperpolarization in sustained mVGNs. Each component was reduced by the estimated 4,9-ah-TTX block: $Na_V$R and $Na_V$P by 90% and $Na_V$T by 70%. Reducing ("blocking") components in various combinations shows that blocking $Na_V$T and $Na_V$P hyperpolarizes resting membrane potential.

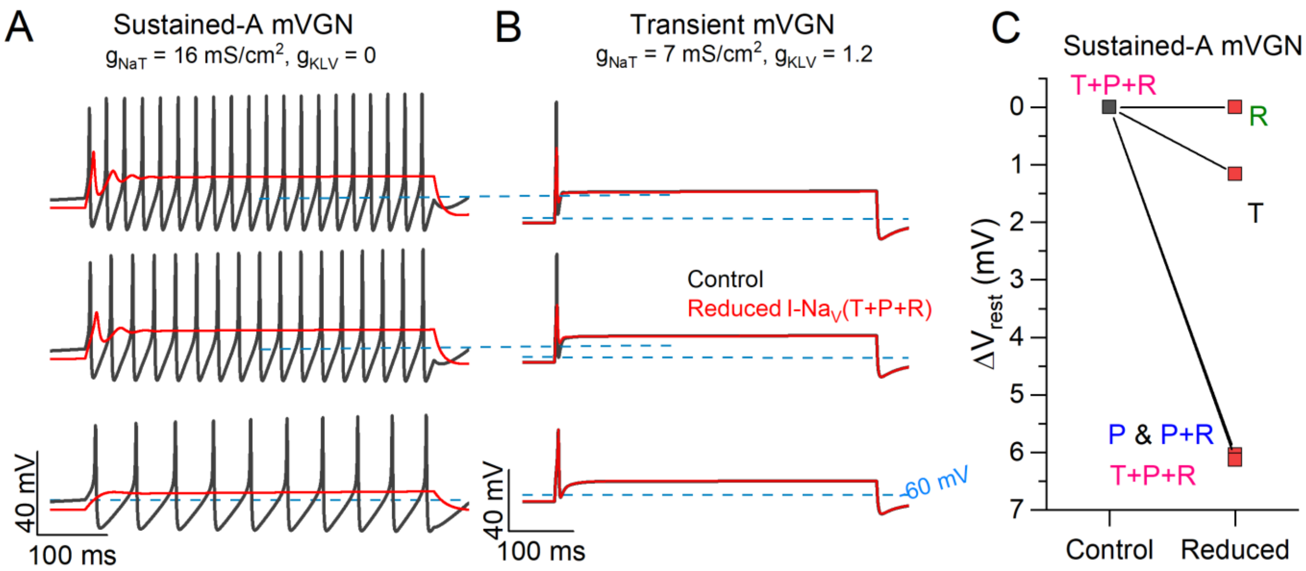

Supplement: Supplementary file 4 [file Image_4.pdf]
