# Supplementary material for: Effects of transient, persistent, and resurgent sodium currents on excitability and spike regularity in vestibular ganglion neurons
Source: Front Neurol. 2024 Nov 18;15:1471118. doi: 10.3389/fneur.2024.1471118 (PMC11608953; doi:10.3389/fneur.2024.1471118)

Supplementary Figure

Figure S5 *Increasing I-Na_VP further increases spike rate in model sustained-A and transient VGNs.*

(A & B) Firing patterns for a sustained-A (A) and transient (B) mVGNs with I-Na_VT density of 16 mS/cm^2 with different amounts of I-Na_VP: 0% (control, black), 3% (red), 5% (blue), 8% (green) and 10% (purple).

(C & D) Summary of spike rates for sustained-A and transient mVGN under each level of I-Na_VP. Increasing I-Na_VP increased spike rate in both mVGNs.

(E & F) A summary of spike train regularity as measured by CV. Increased I-Na_VP had no effect on CV in either mVGN.

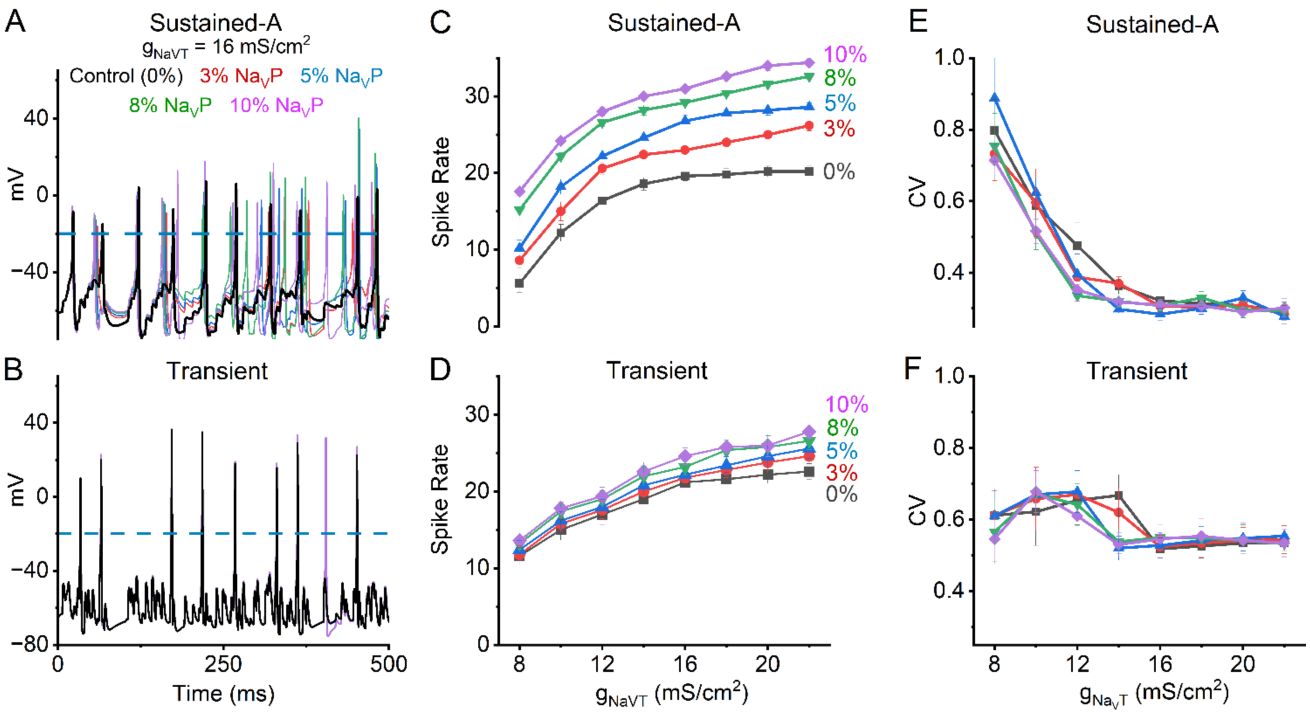

Supplement: Supplementary file 5 [file Image_5.pdf]
