# Supplementary material for: Effects of transient, persistent, and resurgent sodium currents on excitability and spike regularity in vestibular ganglion neurons
Source: Front Neurol. 2024 Nov 18;15:1471118. doi: 10.3389/fneur.2024.1471118 (PMC11608953; doi:10.3389/fneur.2024.1471118)
Supplement: Supplementary file 6 [file Table_1.pdf]

Supplementary Table

Supplementary Table S1

Table S1: Summary of one-way ANOVA tests for voltage clamp experiments					
Na_V conductance density (Fig 2B)		F(2,70) = 6.7, p = 0.002, power = 0.90			
		p		effect size, interpretation	
	Na_VT vs Na_VT+P	0.33			
	Na_VT vs Na_VT+P+R	0.002		0.27, small	
	Na_VT+P vs Na_VT+P+R	0.02		0.41, med	
		**Activation, $V_{1/2}$**		**Inactivation, $V_{1/2}$**	
Na_V current modes (Fig 2C)		F(2,27) = 5.9, p = 0.008, power = 0.83		F(2,26) = 0.38, p = 0.68, power = 0.10	
	Bonferroni test:	p	effect size	p	
	Na_VT vs Na_VT+P	1		0.66	
	Na_VT vs Na_VT+P+R	0.009	0.75, big	0.86	
	Na_VT+P vs Na_VT+P+R	0.02	0.59, med	0.97	
4,9-ah-TTX (Fig 3B)	(n = 12)	F(2, 35) = 1.1, p = 0.35, power = 0.22		F(2, 35) = 5.5, p = 0.009, power = 0.82	
	Bonferroni test:	p		p	effect size
	Control vs Residual	0.52		0.02	0.69, med
	Control vs Blocked	1		1	
	Residual vs Blocked	0.83		0.02	0.66, med
ATX-II (Fig 4)	(n = 7)	**Activation, $V_{1/2}$**		**Inactivation, $V_{1/2}$**	
	Paired t-test:	p	power	p	power
	Na_VT: Control vs ATX-II	0.34	0.13	0.06	0.48
	Na_VP: Control vs ATX-II	0.12	0.33	-	-
