# Supplementary material for: Effects of transient, persistent, and resurgent sodium currents on excitability and spike regularity in vestibular ganglion neurons
Source: Front Neurol. 2024 Nov 18;15:1471118. doi: 10.3389/fneur.2024.1471118 (PMC11608953; doi:10.3389/fneur.2024.1471118)
Supplement: Supplementary file 7 [file Table_2.pdf]

Supplementary Table

Supplementary Table S2

Table S2: Firing pattern passive properties (one-way 4-factor ANOVA)				
Firing pattern	Current threshold (pA) (n)	V_{rest} (mV)	R_{in} (MΩ)	Cm (pF)
Sustained-A	133.3 ± 16.7 (3)	-60.0 ± 2.2 (6)	1020.0 ± 247.8 (5)	21.9 ± 2.3 (6)
Sustained-B	261.1 ± 26.1 (9)	-64.1 ± 1.8 (13)	852.2 ± 280.6 (9)	16.9 ±1.8 (12)
Sustained-C	295.5 ± 34.7 (11)	-63.5 ± 1.3 (10)	475.3 ± 128.8 (7)	15.1 ± 2.1 (9)
Transient	426.8 ± 33.9 (28)	-65.7 ± 0.9 (33)	464.9 ± 73.8 (20)	16.1 ± 1.1 (31)
p	0.001	0.11	0.08	0.18
power	0.94	0.51	0.56	0.41
