# Supplementary material for: Effects of transient, persistent, and resurgent sodium currents on excitability and spike regularity in vestibular ganglion neurons
Source: Front Neurol. 2024 Nov 18;15:1471118. doi: 10.3389/fneur.2024.1471118 (PMC11608953; doi:10.3389/fneur.2024.1471118)
Supplement: Supplementary file 8 [file Table_3.pdf]

Supplementary Table

Supplementary Tables S3

Table S3: Direct comparisons of AP waveform differences (one-way 4-factor ANOVA)

	Current threshold (pA)		Spike height (V_{AP}) (mV)		Peak dV/dt (mV/ms)		AHP (mV)	
	p	Effect size	p	Effect size	p	Effect size	p	Effect size
Sustained-B vs Sustained-A	0.35		0.08		0.79		**0.009**	0.6
Sustained-C vs Sustained-A	0.58		0.05		0.31		**0.0005**	0.9
Sustained-C vs Sustained-B	0.95		0.98		0.71		0.46	
Transient vs Sustained-A	**0.01**	0.4	**0.004**	**0.6**	**0.01**	0.5	**0.000006**	1.0
Transient vs Sustained-B	**0.03**	0.4	0.66		**0.03**	0.3	**0.04**	0.5
Transient vs Sustained-C	0.08		0.92		0.58		0.77	
