# Supplementary material for: Effects of transient, persistent, and resurgent sodium currents on excitability and spike regularity in vestibular ganglion neurons
Source: Front Neurol. 2024 Nov 18;15:1471118. doi: 10.3389/fneur.2024.1471118 (PMC11608953; doi:10.3389/fneur.2024.1471118)
Supplement: Supplementary file 9 [file Table_4.pdf]

Supplementary Table

Supplementary Table S4

**Table S4: Pharmacological effects on AP waveform (paired t-test)**								
**4,9-ah-TTX**	V_{rest} (mV) Transient	V_{rest} (mV) Sustained	Time-to-peak (ms)	Spike Width (ms)	AHP (mV)	V_{AP} (mV)	Voltage threshold (mV)	Peak dV/dt (mV/ms)
Control (n = 12)	-59.5 ± 1.5	-57.6 ± 3.3	4.1 ± 0.3	2.9 ± 0.5	0.0 ± 1.9	74.4 ± 6.4	-42.3 ± 2.3	102 ± 20
Drug	-63.6 ± 2.9	-66.8 ± 3.4	4.8 ± 0.4	3.9 ± 0.6	6.8 ± 1.2	55.1 ± 7.0	-43.3 ± 3.2	64.4 ± 20
p	0.16	**0.03**	**0.05**	0.13	**0.006**	**0.01**	0.90	**0.03**
Effect size		0.67	0.42	0.19	0.72	0.49	0.05	0.41
Power	0.27	0.67	0.53	0.31	0.86	0.76	0.05	0.62
**ATX-II**	V_{rest} (mV)							
Control (n = 7)	-59.2 ± 0.9		3.8 ± 0.3	1.2 ± 0.1	4.8 ± 0.8	92.7 ± 5.9	-49.7 ± 2.1	229 ± 30
Drug	-64.0 ± 2.2		5.5 ± 0.8	2.4 ± 0.6	4.1 ± 1.5	92.8 ± 6.3	-52.2 ± 3.5	209 ± 50
p	0.07		0.07	0.09	0.58	0.97	0.60	0.41
Effect size	0.83		0.45	0.35	0.10	0.01	0.23	0.14
Power	0.45		0.45	0.41	0.07	0.05	0.07	0.11
