# Supplementary material for: Effects of transient, persistent, and resurgent sodium currents on excitability and spike regularity in vestibular ganglion neurons
Source: Front Neurol. 2024 Nov 18;15:1471118. doi: 10.3389/fneur.2024.1471118 (PMC11608953; doi:10.3389/fneur.2024.1471118)
Supplement: Supplementary file 10 [file Table_5.pdf]

Supplementary Table

Supplementary Table S5

Table S5: AP waveform differences between model VGNs											
**mVGN (Figure 8)**			**1st AP:**					**ISI (ms)**	**2nd AP:**		
	Nav modes	Vrest (mV)	Time-to-peak (ms)	ΔV_{AP} (mV)	Spike width (ms)	Peak dV/dt (mV/ms)	AHP (mV)		Time-to-peak (ms)	ΔV_{AP} (mV)	Spike width (ms)
**Sustained-A**	T	-59.6	2.6	96	1	143.4	-6.9	8.6	6.4	69.2	1.4
	T+P	-54.7	2.3	77.9	1.2	100.6	-9.6	8.3	6	61.8	1.4
	T+R	-59.6	2.6	96	1	143.4	-3.3	7.2	5.1	63.7	1.6
	T+P+R	-54.7	2.3	77.9	1.2	100.6	-9.4	8.2	5.9	61.8	1.5
	T+	-59.6	2.5	101.6	0.9	162.4	-7.3	8.6	6.2	74	1.3
**Sustained-B**	T	-61.7	3.9	85.9	1.2	105.1	-5.6	12.9	10.3	57	2.3
	T+P	-61.1	3.8	84.7	1.2	106	-6.1	12.4	9.8	58.8	1.8
	T+R	-61.7	3.9	85.9	1.2	105.1	-5.4	12.8	10.2	57.1	2.3
	T+P+R	-61.1	3.8	84.7	1.1	106	-6	12.3	9.7	58.9	2
	T+	-62.7	3.8	92.7	1.1	123.1	-5.3	12.7	10.2	63.7	1.9
**Sustained-C**	T	-65.2	5.1	86.6	1.4	92.8	-0.8	16.6	13.5	48.5	3.4
	T+P	-64.7	4.9	86.5	1.5	94.2	-1.2	15.9	12.7	51.2	3.2
	T+R	-65.2	5.1	86.6	1.4	92.8	-0.7	16.5	13.4	48.8	3.8
	T+P+R	-64.7	4.9	86.4	1.5	94.2	-1.2	15.7	12.1	51.5	3.2
	T+	-65.2	5	91.9	1.5	108.7	-1.5	16.1	12.9	53.7	2.8
**Transient**	T	-64.3	2.5	77.6	1.5	77.9	5.4				
	T+P	-64.2	2.5	77.9	1.5	79.7	5.3				
	T+R	-64.2	2.5	77.9	1.5	77.9	5.3				
	T+P+R	-64.2	2.5	77.9	1.5	79.7	5.3				
	T+	-64.3	2.5	82	1.4	90.9	4.7				
