# Supplementary material for: Effects of transient, persistent, and resurgent sodium currents on excitability and spike regularity in vestibular ganglion neurons
Source: Front Neurol. 2024 Nov 18;15:1471118. doi: 10.3389/fneur.2024.1471118 (PMC11608953; doi:10.3389/fneur.2024.1471118)
Supplement: Supplementary file 11 [file Table_6.pdf]

Supplementary Table

Supplementary Table S6

Table S6: Effects of I-Na_VP and I-Na_VR on spike rate and CV in model VGNs (two-way 4-factor ANOVA)

		Spike Rate (sp/s)			CV		$CV_{20sp/s}$	
		μ ± SEM	vs. Na_VT p	g	μ ± SEM	vs. Na_VT p	μ ± SEM	vs. Na_VT p
**Sustained-A**	Na_VT	16 ± 0.9	-		0.42 ± 0.03	-	0.45 ± 0.03	-
	Na_VT+R	16 ± 1.1	1		0.45 ± 0.03	1	0.45 ± 0.03	1
	Na_VT+P	23 ± 1.2	**<0.0001**	**0.6**	0.36 ± 0.03	0.08	0.35 ± 0.03	<0.0001
	Na_VT+R+P	24 ± 1.5	**<0.0001**	**0.6**	0.45 ± 0.03	0.96	0.32 ± 0.03	<0.0001
**Transient**	Na_VT	15 ± 0.7	-		0.60 ± 0.02	-	0.60 ± 0.02	-
	Na_VT+R	16 ± 0.9	0.06		0.79 ± 0.03	<0.0001	0.81 ± 0.03	<0.0001
	Na_VT+P	16 ± 0.8	0.2		0.57 ± 0.02	1	0.59 ± 0.02	1
	Na_VT+R+P	18 ± 1.0	<0.0001	0.2	0.79 ± 0.03	<0.0001	0.80 ± 0.03	<0.0001
